# Index Development to Comprehensive Assess Liver Function during the Dairy Cows’ Transition Period in Low-Tropic Conditions

**DOI:** 10.3390/ani14142056

**Published:** 2024-07-13

**Authors:** Rómulo Campos-Gaona, Adriana Correa-Orozco, Arcesio Salamanca-Carreño, Mauricio Vélez-Terranova

**Affiliations:** 1 Facultad de Ciencias Agropecuarias, Universidad Nacional de Colombia, Sede Palmira, Palmira 763531, Colombia; 2 Facultad de Medicina Veterinaria y Zootecnia, Universidad Cooperativa de Colombia, Villavicencio 500001, Colombia

**Keywords:** bovine, hepatic function index, metabolism, transition period

## Abstract

**Simple Summary:**

The physiological changes during the peripartum, especially during the transition period, generate metabolic and endocrine alterations that affect the health, reproductive efficiency and productive potential of dairy cows in the tropical environment. In order to generate tools to study the homeostatic dynamics, a proposal was developed to integrate metabolic, endocrine, productive variables and changes in body condition into a single index. It has been demonstrated that some indexes are valid and useful for knowing the hepatic activity of the postpartum gluconeogenic axis.

**Abstract:**

The aim of this work was to develop a liver tissue function index during the transition period of dairy cows managed in low-tropic conditions. In two farms, twenty crossbred and synthetic native cows during the peripartum period were selected, and blood samples were taken on days −30 and −15 prepartum, the calving day, and 7, 20, 35, 50, 65, 80 and 105 days postpartum for serum metabolic tests. On each measurement day, body condition scores (BCS) and parameters on nitrogen metabolism (total protein—TP, albumin—ALB, globulin—GLOB, urea), adipose tissue metabolism (cholesterol—COL, non-esterified fatty acids—NEFA) and two transaminases (alanine aminotransferase—ALT and aspartate aminotransferase—AST) were evaluated. Data analysis included the Spearman correlation, principal components, multiple linear regression and cluster analysis. Results showed that regarding the days after calving and BCS, a liver tissue function index can be constructed using the TP, urea, COL, ALT and NEFA. The estimated index generated three groupings, both by days after calving and BCS. In the former, the index discriminated the metabolic behavior in the prepartum, parturition and postpartum periods, while in the latter, the index discriminated between extreme (2.25, 2.50 and 4.25), slightly low (2.75 and 3.0) and slightly high (3.25 to 4) conditions. The results allow us to conclude that it is feasible to construct mathematical function indexes for liver function to monitor metabolic changes during highly demanding productive phases in dairy cows under tropical conditions.

## 1. Introduction

The multiple hepatic functions are fundamental for cow’s milk production [1], especially during the period between three weeks before and three after calving, also called the transition period [2]. Among them, those related to energy metabolism and its effects on the hepatic tissue response to carry out gluconeogenesis and synthesis of urea, insulin-like growth factor type 1 (IGF1) [3], acute phase proteins associated with inflammatory responses and other transport proteins also called negative proteins [4]. Likewise, the hepatic effect on reproductive function [5] and postpartum ovarian reactivation [6], or animal sanitary status [7], has also been studied. On the other hand, several metabolites have been proposed to analyze the main functions of hepatic synthesis [8,9]. In recent years, biomarkers other than liver transaminases have been used to improve the accuracy of diagnostic techniques [10].

Several studies have characterized the liver as an integrative organ that controls several vital functions of energy and nitrogen metabolism, the oxidation of a wide metabolites variety to regulate the energy supply and performs hepatic protein synthesis, as an integrative mechanism of homeostasis and homeorhesis processes [1,11].

In ruminants, liver function presents special metabolic considerations, since volatile fatty acids are generated from ruminal fermentation, which, when absorbed, constitute the main oxidative and gluconeogenesis fuel (especially propionate). In these circumstances, fermentable substrates alter energy intake and, in turn, fermentation modifies feed intake patterns [12].

In high-producing dairy cows, metabolic alterations during the transition period occur; in this way, a timely prediction of liver function in cows peripartum will help in the prevention and treatment of pathologies, which usually affect profitability and cause premature animal culling rate [13]. In their liver, aspartate aminotransferase (AST), alanine aminotransferase (ALT) and gamma-glutamyltransferase (GGT) enzymes show high activity and are assayed for monitoring acute or chronic function or impairment. Determination of AST and ALT in dairy cows is associated with hepatic steatosis, low appetite and ketosis occurrence during early lactation. Increased serum AST activity is a sensitive marker of liver damage in several species, but it is not absolutely valid in ruminants, and for this reason, a comprehensive liver function test is preferred [10].

The construction of associative models as hepatic indexes has been sought in order to predict the physiological state, potential or alteration of dairy cows’ productive ability [8,13,14]. Some authors proposed an index to quantify liver activity, in cases of possible inflammatory processes; for this, total protein, albumin and bilirubin were employed in the search for biomarkers that could relate to the liver status [15]. To comprehensively evaluate the changes associated with the transition period, other indexes have been used with relative success, or have been employed in behavioral evaluations during this productive phase [16,17,18]. The major loss of homeostasis and metabolic alteration occurs during the negative energy balance status (NEBAL), so this parameter has also been used in serial evaluations [19,20]. Likewise, these indexes were used for cross-sectional studies in situations of associated morbidity, such as ketosis, hypocalcemia and fatty liver [21,22].

Based on the evidence on health and production assessment tools, and as a contribution to the exploration of metabolic disease monitoring strategies, the objective of this work was to develop a liver tissue function index during the transition period of dairy cows managed in low-tropic conditions.

## 2. Materials and Methods

### 2.1. Study Region

In this project, information from two farms under different management conditions located in the low-tropic agroecological zones (Valle del Cauca, Colombia) was used. The farms were located between 3′30 to 4′10 N and 76′21 to 76′46 W, ecologically corresponding to a tropical dry forest zone according to Holdridge’s classification of life zones [23].

Farm 1 corresponded to a specialized dairy system with Gyr × Ayrshire and Gyrolando crossbred animals, managed under rotational grazing, in pastures of star grass (*Cynodon mfluensis*). Animals with more than three calvings and an average milk production of 15 L/day were used in the study.

Farm 2 corresponded to an intensive silvopastoral farm with a synthetic creole breed (Holstein 40%, Shorthorn 30% and Hartón del Valle 30%) named Lucerna, grazing star grass (*Cynodon mfluensis*) and leucaena (*Leucaena leucocephala)* forages. Animals with an average milk production of 10 L/day and with more than three calvings were included in the study. In both farms, water and salt were supplied ad libitum and balanced feed was given according to milk production at a rate of one kilogram of supplement for four liters of milk produced.

### 2.2. Animals and Sampling Scheme

The experiment was carried out under a randomized complete block design, considering the farm as a blocking factor. In each system, ten pluriparous cows close to calving were randomly selected. The sample size corresponded to the minimum animal number allowed for metabolic indicators studies [24]. On days −30 and −15 of prepartum, the day of calving (0), and on days 7, 20, 35, 50, 65, 80 and 105 postpartum. Blood samples were obtained by coccygeal venipuncture using vacuum silicone tubes and a multichannel needle. The tubes were transported under refrigeration (4 °C) to the processing site within 35 to 75 min. The tubes were centrifuged at 1160 G for 15 min, and the serum was removed and fractionated into aliquots that were frozen at −20 °C until analysis, which was performed in duplicate. Acusera^®^ control serum, Bovine Chemistry Assayed Level 2 (Randox, Crumlin, Ireland), was used as an internal control. In serum, the following parameters were determined using commercial reagents (Randox) for colorimetric enzymatic techniques: non-esterified fatty acids (NEFA), cholesterol (COL), urea, total protein (TP), albumin (ALB), globulins (GLOB), aspartate aminotransferase (AST) and alanine aminotransferase (ALT). Analyses to determine metabolite and enzyme concentrations were performed by colorimetric enzyme assays. Specific commercial reagents and a reading semi-automatic analyzer (Rayto^®^, Shenzhen, China) were used. The analyzed metabolites were selected with the intention of evaluating nitrogen metabolism (TP, ALB, GLOB, urea), adipose tissue metabolism (COL and NEFA) and two transaminases—ALT and AST—indicative of hepatocyte health status [8,21]. Urea allows us to evaluate the detoxifying synthesis of nitrogenous compounds, while the negative energy balance, was indirectly reflected in the NEFA concentration. 

On each sampling day, the body condition score (BCS) was also evaluated by the same person during the experiment, using the standard 1–5 scale, where 1 corresponded to emaciated animals and 5 to obese animals [25].

### 2.3. Statistical Analysis

To construct the index, in the first phase, Spearman correlations among the eight metabolic variables (PT, ALB, GLOB, urea, COL, NEFA, AST and ALT) were estimated, with the aim of identifying highly associated variables (>0.80) and select some of them for further analysis, avoiding multicollinearity. Afterwards, a principal component analysis (PCA) with the standardized metabolic variables was performed, using sampling periods (−30 prepartum to 105 postpartum) as classification criteria. The variables with the highest weights (eigenvectors) in the first principal component (PC1) were selected to perform a second PCA under the same conditions beforementioned. This procedure was carried out with the intention of reducing the number of variables under study and selecting a linear combination of variables that would explain the greatest proportion of the data variability. Since PC1 of the second PCA explained a greater variation percentage in the data, the adjusted values of this component in each animal were used as an index of liver function (I-PC1). The behavior of the I-PC1 was related to the animals’ BCS and days after calving. 

To facilitate the estimation of the I-PC1 index, a multiple linear regression equation was adjusted, using the metabolic indicators that most contributed to the PC1 from the second PCA as regressor variables, and the response variable was the I-PC1 index. To identify the variables with the greatest predictive capacity of the index, the backward variable elimination method was employed with a variable retention criterion of *p* < 0.05. Additionally, to improve the fit of the estimated equation, quadratic trends of the response variable (I-PC1) were analyzed with respect to each of the predictors, through the graphical analysis of partial residuals. The fit capacity of the estimated equation was evaluated by leave-one-out cross-validation, using the following parameters:
RMSE$\sqrt{\frac{\sum (x_{i}-x_{p}{)}^{2}}{n}}$MAE$\frac{{\sum |x}_{i}-x_{p}|}{n}$R^2^$\frac{\sum (x_{i}-x_{p}{)}^{2}}{\sum (x_{i}-\bar{x}{)}^{2}}$RMSE: root mean square error. MAE: mean absolute error. R^2^: coefficient of determination. $x_{i}$: observed value. $x_{P}$: predicted value and n: number of observations. $\bar{x}$: index mean value

The predicted index values (P-CP1) were grouped through hierarchical cluster analysis, using days after calving and/or BCS as classification criteria. This was carried out with the intention of identifying the days after calving and BCS where the index behaved similarly. The ‘average linkage’ classification algorithm and Euclidean distance were used in the analyses, and the number of clusters was determined according to the observed index behavior, avoiding the formation of groups with a single sampling period (days after calving) or BCS. Finally, descriptive statistics were estimated within each cluster to observe the behavior of the index and the metabolites used in its construction.

Subsequently, the predicted P-PC1 index was compared with a theoretical index called HEpINdex, obtained from a modification of the hepatic index [15]. For the construction of the theoretical HEpINdex, the same metabolites used to construct the I-PC1 index were used (TP, ALT, urea, NEFA and Col). Each metabolite data from each animal was standardized using the respective average and general standard deviation obtained during the ten sampling periods and, subsequently, the contribution of each metabolite was added to estimate the final value of the theoretical HEpINdex. The relationship between both indexes was analyzed by Spearman’s correlation coefficient (*p* < 0.05). Also, the P-PC1 and the theoretical HEpINdex were grouped according to the previous clusters formed, and statistical descriptive measures with respect to days after calving and BCS were estimated. The analyses were performed with the Infostat software, version 2020 [26] and the model cross-validation was conducted using RStudio version 4.3.1. [27] and the caret package.

## 3. Results

**Figure 1:**
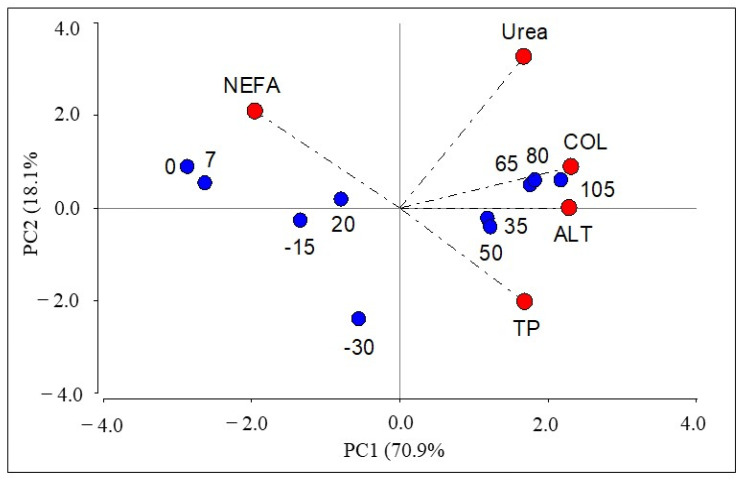
Biplot of the first two principal components of the second PCA performed with the variables with the highest eigenvectors (NEFA, TP, ALT, COL and urea). Red points: variables, blue points: days after calving, PC: principal component.

Spearman’s correlation analysis indicated that among the variables under study, only TP and GLOB presented a high correlation (r = 0.89). To avoid multicollinearity, the TP variable was selected for the following analyses, since, within the metabolic indicators parameters, TP is considered an adequate general reference of nitrogen metabolism [28]. Thus, the first PCA was performed considering seven variables (TP, ALB, urea, COL, NEFA, AST and ALT), and the results showed that PC1 explained 51% of data variability, with metabolic variables like TP, urea, COL, NEFA and ALT presenting the highest weights. The second PCA with the five aforementioned variables showed that PC1 went on to explain 71% of data variability, which implied a 20% increase in the explained variance. The biplot of the first two principal components of the second PCA is shown in Figure 1.

**Figure 2:**
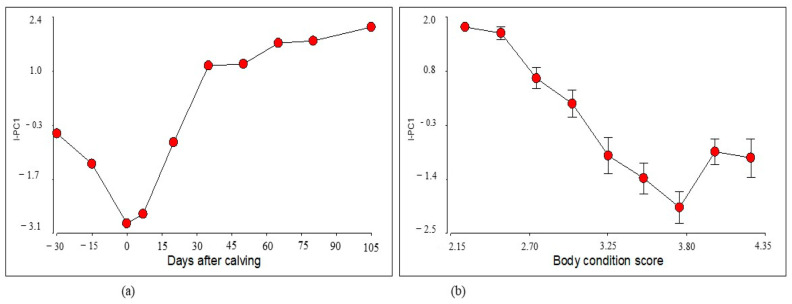
Relationship between the estimated I-PC1 index with the days after calving (**a**) and the animals’ body condition score (**b**).

It can be observed how PC1 groups the prepartum, calving and 20-day postpartum periods, and separates them from the rest of the periods corresponding to days 35 to 105 postpartum. Considering that PC1 allows the separation of the evaluated periods and explains the largest proportion of data variability, only this component and its linear combination were used to construct the index I-PC1, which presented the following form: I-PC1 = 0.38 * TP + 0.37 * urea + 0.52 * Col + 0.51 * ALT − 0.44 * NEFA. The behavior of the I-PC1 index in relation to days after calving and BCS is shown in Figure 2.

**Figure 3:**
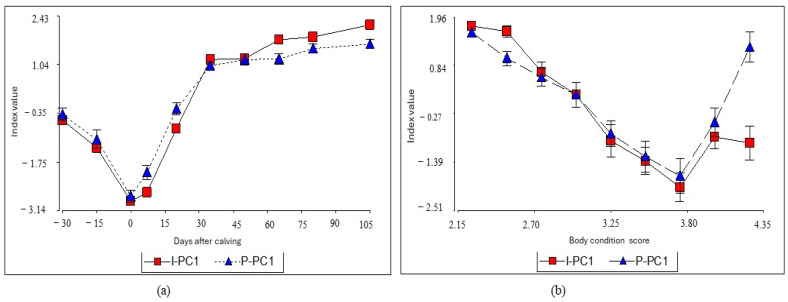
Relationship between the estimated index (I- PC1) and the predicted index (P-PC1) by the multiple regression model on different days after calving (**a**) and animals’ body condition scores (**b**).

Given that the estimation of the I-PC1 index from the linear combination obtained with the PCA cannot be performed directly, since it is first necessary to standardize the variables by the evaluated periods, implies that its estimation is not practical, which would limit its use. Thus, to facilitate the construction of the index, a multiple linear regression was adjusted to predict the I-PC1 from the most representative variables of the PCA (PT, urea, COL, NEFA and ALT). The results showed that the I-PC1 index could be predicted with the following regression equation: P-PC1= −7.03 + 0.02 * PT + 0.12 * urea + 2.32 * COL − 0.17 * COL^2^ − 2.50 * NEFA (R^2^ = 0.82, RMSE = 0.76, MAE = 0.59). According to the leave-one-out cross-validation analysis, the performance of the estimated regression model is consistent, as its predictive ability remained high and with a low error margin (R^2^ = 0.80, RMSE = 0.79 and MAE = 0.60). The relationship between the I-PC1 obtained and that predicted from the regression model (P-PC1) is shown in Figure 3.

According to the cluster analysis, three groupings were enough to describe the behavior of the P-PC1 index for both, days after calving and BCS (Table 1).

**Figure 4:**
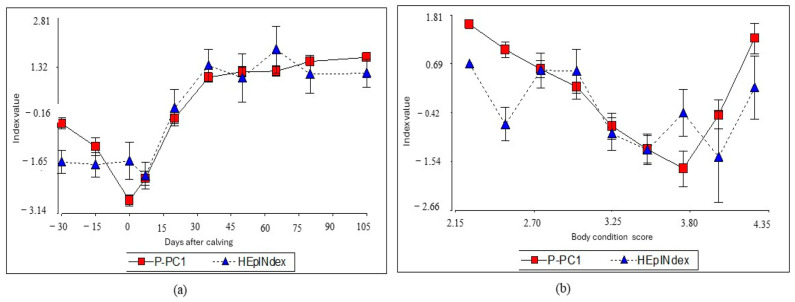
Comparison between P-PC1 and the theoretical index (HEpINdex) in different days after calving (**a**) and BCS (**b**) as indicators of liver function in dairy cows during the transition period.

Figure 4 shows the comparison between the P-PC1 and the theoretical HEpINdex index obtained by the modified version of the hepatic index [15] is shown in Figure 4. The correlation between the indexes was 0.64 (*p* < 0.0001), a relatively high value, suggesting that both indexes behaved similarly. To deepen the comparison between indexes, the performance of both in the clusters previously formed days after calving and BCS is shown in Table 2.

## 4. Discussion

The PCA analysis allowed us to identify groups of periods with similar behavior in terms of NEFA, TP, ALT, COL and urea metabolites during the peripartum of dairy cows in tropical environments. The prepartum, calving and first 20 days postpartum periods were similar and characterized by high NEFA concentrations. NEFA shows lipid metabolism resulting from loss of body condition score associated with lower energy intake due to low dry matter intake [9]. These periods are separate from those found from 35 to 105 days postpartum, which were characterized by higher Col, ALT, urea and TP concentrations. From a biological point of view, this is an expected separation, since it defines a behavior closely related to the physiological transition period, where homeostatic changes occur and allow the stability of metabolic challenges [29].

The NEFA, TP, ALT, COL and urea metabolites allowed us to explain the largest proportion of data variability and separate the transition period. The TP and urea are related to nitrogen metabolism, while NEFA and COL are involved in energy lipid metabolism. These two metabolic pathways are important in milk-producing animals, because of the extractive demand for nutrients occurring in the mammary gland [1,30]. These metabolites associated with liver function integrally incorporate diverse biochemical pathways occurring in liver tissue, which, in turn, allow the indirect evaluation of possible metabolic responses in animals and thus predict productive alterations, as has been proposed by other authors [8,31,32]. Given the complex and diverse functions developed in the liver, it is a constant challenge to find tools to assess function and predict productive behavior beyond pathological situations. This difficulty relies on the fact that their accuracy and reliability respond to clinical needs, which are different from those of possible productive scenarios [33].

### 4.1. I-CP1 Index Performance

Figure 2 shows the I-PC1 index behavior with respect to the days after calving and the BCS of the animals. Regarding the days after calving, the I-PC1 index presents a significant decrease around calving and seven days postpartum, indicating that the homeostatic maladjustment originated by calving and lactation affects the index values; in the same sense, the index proposed by Batista et al. [34] is positive and distances itself from the zero value, as the homeostatic and homeorhetic functions are regulated. This situation is observed in the present work from the twenty days postpartum onwards and continues to increase until it stabilizes after the fifty days postpartum when milk production has passed its maximum production point.

The behavior of the I-PC1 index with respect to BCS showed that the lowest values are observed when BCS increases, and it becomes erratic in conditions above 4 (on the 1–5 scale). This could be due to the accumulation and metabolization of lipids, which significantly alters the liver function efficiency. It is known that high BCS is associated with metabolic alteration of fatty liver [14].

Although the I-PC1 index seems to present biological meaning with respect to the transition period and BCS, its estimation was not easy, so it was necessary to build a mathematical model that allowed a quick estimation. The relationship between the estimated I-PC1 and predicted P-PC1 indexes is shown in Figure 3. The predicted and observed values are similar among the different days after calving and BCS. The coefficient of determination was high, indicating that 82% of the variation of I-PC1 is explained by the regression model. In addition, the leave-one-out cross-validation analysis indicated that model performance was consistent with a low error margin. These results suggest that the estimated model can be used to predict the I-PC1 index acceptably, and also, could be useful in other production scenarios with similar conditions to the present study, in order to estimate the index and assess the liver function of dairy cows during the transition period.

In general, the predicted index (P-PC1) behaves as expected during the studied periods and BCS. However, in the latter, an erratic behavior was observed with BCS equal to 4.25, which can be attributed to the fact that animals in the peripartum and first phase of lactation unusually exhibit body conditions higher than 3.5. In the data, BCS higher than 4.0 were infrequent, which could affect the proper adjustment of the prediction algorithm.

### 4.2. Index Clustering and Performance

The cluster analysis formed three groups for both days after calving and BCS (Table 1). In the case of days after calving, the first group consisted of the prepartum (−30 and −15 days) and 20 days postpartum periods, with an average index value of −0.56; metabolite concentrations did not show significant changes in this grouping. The second group was formed by the calving and seven days postpartum, with an average index value of −2.38. In this group, a greater homeostatic maladjustment is evidenced, where high NEFA values indicate a greater energy deficit, associated with negative energy balance (NEBAL), derived from the beginning of milk synthesis [35]. The third group was made out of the periods from day 35 to 105 postpartum, with an average index value of 1.3. The NEFA concentration shows that the NEBAL has been exceeded since the values in this group decreased. Likewise, the urea concentration is raised, which normally occurs as dry matter intake increases in the postpartum period [2].

The clustering conformed with respect to BCS indicated that the first group was formed by extreme BCS (2.25, 2.50 and 4.25), with an average index value of 1.09. In the second group, 2.75 and 3.0 BCS were grouped, with an average index of 0.40. Finally, the third group included BCS between 3.25 to 4.0, with an average index value of −1.04 (Table 1). Compared with other proposals, the BCS found in the first group would indicate homeostasis, which cannot be explained (index value positive) [34], since the BCS in the first group normally presented the greatest oscillations between high and low BCS as a consequence of metabolic and endocrine imbalances, with both situations determined as undesirable from a physiological point of view and of the metabolic dynamics in the peripartum [25].

The behavior of the P-CP1 index was also compared with a theoretical index called HEpINdex, derived from the Bertoni and Trevisi proposal [15] (Figure 4). Both indexes behaved similarly, reflecting an acceptable correlation coefficient of 0.64. The metabolites analyzed also compared the behavior of both indexes in the three previously established groupings obtained in the cluster analysis (Table 2). Among days after calving, the indexes varied between −2.38 and 1.3 and −1.76 and 1.3 for P-CP1 and HEpINdex, respectively, while, regarding BCS, the variation range is −1.04 to 1.09 and −098 to 0.53 for P-PC1 and HEpINdex, correspondingly. It has been reported that values higher than zero, in the liver function index, indicate a good hepatocyte function [21,34]. The metabolic concentrations found indicate that most of the samples correspond to a physiological liver function. Those index values outside the range would be atypical values susceptible to be considered as situations from homeorhetic difficulties affecting the productive function [29].

The performance of the P-PC1 and HEpIndex indexes, with respect to days after calving, indicated that both indexes showed similar trends, with negative values in clusters 1 and 2, and positive values in cluster 3. However, in relation to the body condition score, both indexes showed positive and negative values in clusters 2 and 3, respectively, which indicates the physiological changes of postpartum. In cluster 1, the value signs were different, influenced by the higher negative value of HEpINdex. The higher the negative value, the greater the imbalance and therefore the lower the homeostatic response [34].

The results of the present study suggest that using complex indicators in the challenging physiological phases facilitates finding tools that allow corrective nutritional, metabolic or other actions to respond to the productive challenges. Nevertheless, the profound physiological changes during the transition period and the multiple environmental challenges make the conceptual process a difficult task for modeling [8,15].

The basic construction of the P-PC1 index developed from the TP, urea, COL, NEFA and ALT metabolites tended to present an atypical behavior in some BCS, with a less erratic behavior with respect to the days after calving (Figure 4). The index changed according to the BCS between pre- and postpartum, with low values at low BCS. The observed and predicted behavior of the indexes followed a biologically meaningful dynamic, which, in a practical sense, allows them to be used as a predictive strategy for homeostatic adjustment behavior. In cluster three, which grouped days after calving from 35 to 105, the index values were positive, while in the peripartum and first lactation phase, negative values were observed, corresponding to the physiological phases with the greatest metabolic challenge [31]. In addition, the basic theoretical HepINdex is useful as a screening indicator of animal behavior in the herd and productive phase, as presented in other indexes [13,16].

Further research is required to validate the usefulness of the obtained indexes (P-PC1 and HEpINdex) under different productive contexts and with animals with different milk production levels. This is required since it is not feasible to achieve a single indicator to study liver function, some diagnostic tests are important in inflammatory or tissue alterations [9] or predictive in productive aspects [22]. Therefore, it is necessary to employ a combination of analytes and monitoring methods to analyze the performance of dairy cows in the transition period [31,36].

## 5. Conclusions

The present work demonstrated the possibility of using mathematical tools in the construction of indexes to estimate physiological functions from the individual response of the animals, through the use of metabolites associated with liver function and the direct monitoring of metabolic changes during the period transition in dairy cows in tropical conditions.

## Figures and Tables

**Table 1:** Average values of the P-PC1 index and the metabolites used in its construction on different days after calving and body condition score.

**Cluster**	**Days after Calving**	**n**	**Mean** **Index**	**TP** **g/dL**	**Urea** **mmol/L**	**COL** **mmol/L**	**NEFA mmol/L**	**ALT** **U/I**
1	−30, −15 and 20	47	−0.56	74.78 ± 6.67	5.57 ± 1.55	3.3 ± 0.88	0.66 ± 0.26	23.15 ± 4.37
2	0 and 7	31	−2.38	69.3± 4.52	5.8± 1.38	2.45± 0.56	0.92± 0.29	19.7± 3.19
3	35 to 105	80	1.30	77.2± 6.67	6.47± 2.15	5.17± 1.23	0.56± 0.18	27.64± 8.04
**Cluster**	**BCS**	**n**	**Mean** **index**	**TP** **g/dL**	**Urea** **mmol/L**	**COL** **mmol/L**	**NEFA mmol/L**	**ALT ** **U/I**
1	2.25; 2.50 and 4.25	14	1.09	72.52 ± 4.26	6.53 ± 1.82	4.47 ± 0.86	0.48 ± 0.07	25.12 ± 4.87
2	2.75 and 3.0	89	0.40	76.19 ± 7.22	6.31 ± 2.16	4.45 ± 1.63	0.64 ± 0.25	25.23 ± 8.3
3	3.25; 3.50; 3.75 and 4.0	50	−1.04	73.06 ± 6.51	5.54 ± 1.04	3.17 ± 1.06	0.74 ± 0.3	23.71 ± 5.31

BCS: body condition score (scale of 1 to 5, where 1 corresponded to emaciated animals and 5 to obese animals). TP: total protein. COL: cholesterol. NEFA: non-esterified fatty acids. ALT: alanine aminotransferase. Values correspond to mean ± standard deviation.

**Table 2:** Average values of the P-PC1 and the theoretical HEpINdex in the constructed clusters by days after calving and body condition score.

		P-PC1			HEpINdex		
Cluster	Days after Calving	Mean	Min	Max	Mean	Min	Max
1	−30, −15 and 20	−0.56	−2.37	1.62	−1.03	−4.43	4.8
2	0 and 7	−2.38	−4.29	−0.74	−1.76	−5.62	2.3
3	35 to 105	1.3	−0.42	2.45	1.3	−3.42	7.06
**Cluster**	**BCS**	**Mean**	**Min**	**Max**	**Mean**	**Min**	**Max**
1	2.25; 2.50 y 4.25	1.09	0.16	2.17	−0.47	−2.62	1.29
2	2.75 and 3.0	0.4	−4.29	2.45	0.53	−5.62	7.06
3	3.25; 3.50; 3.75 and 4.0	−1.04	−4.09	1.47	−0.98	−4.07	2.39

P-PC1: estimated index; HEpINdex: theoretical index; BCS: body condition score (scale of 1 to 5, where 1 corresponded to emaciated animals and 5 to obese animals). Min: minimum value. Max: maximum value.

## Data Availability

Data are available upon reasonable request to the first author.
